# Pacific Coast Fisheries

**Published:** 1881-01

**Authors:** 


### PACIFIC COAST FISHES.

The United States Fish Commissioners have obtained on the Pacific Coast 270 species of fish. Among these are nineteen species of sharks. Two large-toothed man eater sharks, caught in Monterey Bay, measured from 23 to 24 feet in length, and weighed fully two tons each. Another variety of shark found on the coast averages 32 to 33 feet in length and weighs three tons. Their teeth are small and they are not dangerous. Monterey is a middle ground, where the fishes from the north and south meet, and no locality on the Atlantic Coast is so rich in species; San Francisco about the same; Santa Barbara, 95; San Diego, 80, and Puget Sound, 90. The so-called perch, found on the California coast, are not true perch. A million and a half of salmon, averaging from 25 to 30 pounds each, are taken from the Columbia river, and from seven to eight millions of pounds of fish are taken from San Francisco Bay and marketed. — *Scientific American.*

